# Glycemic variability is associated with subclinical atherosclerosis in Chinese type 2 diabetic patients

**DOI:** 10.1186/1475-2840-12-15

**Published:** 2013-01-15

**Authors:** Yifei Mo, Jian Zhou, Mei Li, Yuwei Wang, Yuqian Bao, Xiaojing Ma, Ding Li, Wei Lu, Cheng Hu, Minghua Li, Weiping Jia

**Affiliations:** 1 Department of Endocrinology and Metabolism, Shanghai Jiao Tong University Affiliated Sixth People’s Hospital, 600 Yishan Road, Shanghai, 200233, China; 2 Department of Diagnostic and Interventional Radiology, Shanghai Jiao Tong University Affiliated Sixth People’s Hospital, Shanghai, China; 3 Department of Biochemistry, Microbiology and Immunology, Ottawa Institute of Systems Biology, University of Ottawa, Ottawa, Ontario, Canada

**Keywords:** Glycemic variability, Type 2 diabetes, Atherosclerosis, Intima-media thickness, Magnetic resonance angiography.

## Abstract

**Background:**

The contribution of glycemic variability to macrovascular complications remains unclear. We therefore investigated the association between glycemic variability and cervical and/or intracranial atherosclerosis in Chinese type 2 diabetic patients.

**Methods:**

We conducted a cross-sectional study in 216 type 2 diabetic patients with a hemoglobin A_1c_ of 8.3 ± 1.7% and a median diabetes duration of 9.0 years. The standard deviation of blood glucose values (SDBG) and the mean amplitude of glycemic excursion (MAGE) were calculated from continuous glucose monitoring system data for assessing glycemic variability while 24h mean blood glucose (MBG) was calculated for measuring overall blood glucose level. Magnetic resonance angiography (MRA) was used to detect cervical and/or intracranial plaque, and ultrasonography was used to quantify carotid intima-media thickness (IMT) as an index of subclinical atherosclerosis.

**Results:**

One hundred and fifty-three patients (70.8%) presented with cervical and/or intracranial lesions on MRA among 216 patients in the study. Elder age, increased systolic blood pressure, increased MBG and elevated low density lipoprotein cholesterol were independent contributors to plaque formation. In patients without stenosis (*n* = 63), SDBG (*r* = 0.412, *P* = 0.001) and MAGE (*r* = 0.365, *P* = 0.005) were both correlated with carotid IMT and these relationships remained significant in multiple linear regression analysis (multiple *R*^*2*^ = 0.314 for the model including SDBG and multiple *R*^*2*^ = 0.268 for the model including MAGE). However, SDBG and MAGE were not significantly different among patients with different stenosis degrees.

**Conclusions:**

Glycemic variability is associated with subclinical atherosclerosis in Chinese type 2 diabetic patients.

## Background

Macrovascular diseases such as coronary artery disease and cerebrovascular disease are major complications of type 2 diabetes. Previous studies have shown the association between averaged mean levels of glycemia and macrovascular complications [1,2]. Meanwhile, several lines of evidence have raised the possibility that glycemic variability, which includes both upward and downward acute glucose changes, is another important component of dysglycemia [3]. Glycemic variability can have deleterious effects on the development and progression of macrovascular complication through oxidative stress and endothelial dysfunction [4,5]. With the emergence of continuous glucose monitoring (CGM) technology, the assessment of glycemic variability is now much less cumbersome. However, the exact role of glucose variability in the development of atherosclerosis remains unclear.

Atherosclerosis is a complex multifactorial disease, often preceding the development of diabetic macrovascular complications. Carotid intima-media thickness (IMT), measured noninvasively by ultrasonography, is accepted as an indicator of subclinical atherosclerosis, and has been reported to be directly associated with an increased risk of cardiovascular and cerebrovascular diseases [6]. Furthermore, in terms of defining the presence of plaque, the technology of 3D time-of-flight magnetic resonance angiography (3D-TOF-MRA) and contrast-enhanced magnetic resonance angiography (CEMRA) are reported to be more informative than ultrasonography [7,8]. To gain insight into the role of glycemic variability in the different stages of atherosclerosis, we used both ultrasonography and MRA to determine preclinical atherosclerosis and plaque formation. The aims of this study were: (i) to investigate the prevalence of atherosclerotic plaque by examining the cervical and intracranial arteries using MRA in Chinese type 2 diabetic patients; and (ii) to explore the role of glycemic variability in both the subclinical stage and plaque formation stage of atherosclerosis in this population.

## Methods

### Study populations

The study was carried out over a period of 12 months between 1 January 2008 and 31 December 2008. We included 216 type 2 diabetic patients who consulted for complications screening at the Department of Endocrinology and Metabolism in our hospital on an annual basis. Type 2 diabetes was diagnosed according to 1999 World Health Organization (WHO) criteria [9]. Eligibility was based on a stable therapeutic regimen with oral hypoglycemic agents and/or insulin for the previous 3 months. Exclusion criteria included recent acute complications such as diabetic ketoacidosis and hyperglycemic hyperosmolar state, severe and recurrent hypoglycemic events in the previous 3 months, a history of hepatic or renal impairment, or of other diseases that can influence glucose metabolism, including recent acute cerebral stroke, acute myocardial infarction, malnutrition, and cancers. In addition, claustrophobic patients, and patients with valvular prostheses, vascular clips, cardiac pacemakers, or other implanted devices sensitive to strong magnetic fields, were also excluded from the study. The original study received approval from the Ethics Committees of Shanghai Jiao Tong University Affiliated Sixth People’s Hospital in accordance with the principle of the Helsinki Declaration. Written informed consent was obtained from each participant.

### CGM parameters

Subcutaneous interstitial glucose was monitored continuously for 3 consecutive days using a retrospective CGM system (Medtronic Inc, Northridge, CA, USA). The sensor of the CGM system was inserted on day 0 and removed after 72 h; this generated a daily record of 288 continuous sensor values. A minimum of four capillary blood glucose readings per day, as measured by a SureStep blood glucose meter (LifeScan, Milpitas, CA, USA), were entered into the CGM system for calibration. The 24h mean blood glucose (MBG) level was calculated from the 288 consecutive sensor readings over a 24h period. The 24h MBG and intraday glycemic variability were based on the mean values taken on days 1 and 2. Intraday glycemic variability parameters include the standard deviation of blood glucose values (SDBG) [10] and the mean amplitude of glycemic excursions (MAGE) [11]. MAGE was calculated by measuring the arithmetic mean of the differences between consecutive peaks and nadirs; measurement in the peak-to-nadir or nadir-to-peak direction was determined by the first qualifying excursion; only excursions of more than 1 SD of the mean glycemic values were considered because MAGE was designed to quantify only major swings of glycemia instead of minor ones. All patients wore blinded CGM system and received the same therapy as before admission. The CGM monitoring was performed in the hospital and the mean CGM period ± SD was 71 ± 3h. The reference values for CGM system in Chinese population have been reported elsewhere [12,13].

Patients were instructed to adhere to a standard diet during the three-day period of CGM sensor monitoring. The diet was designed to ensure a total daily caloric intake of 25 kcal/kg/day, with 55% of calories coming from carbohydrates, 17% from proteins, and 28% from fats. Written instructions were provided to achieve the appropriate caloric content and to guide the consumption times, which included breakfast (20% of daily calories, 06:30–07:30), lunch (40%, 11:30–12:30), and dinner (40%, 18:00–19:00).

### Cervical and intracranial magnetic resonance angiography (MRA) examination

All MRA examinations were performed using a 3.0 tesla MR system (Achieva, Philips Medical Systems). Intracranial MRA was performed using a 3D-TOF-MRA sequence with an 8-channel head coil or a 16-channel craniocervical joint coil. The 3D-TOF-MRA was obtained with repetition time/echo time (TR/TE) 30/3.2 msec, flip angle 20°, field of view (FOV) 250 × 220 mm, four slabs (180 slices), 1.2 mm slice thickness, matrix 1024 × 1024 and an acquisition time of 8 min 56 s. The acquired images were then transferred to a separate workstation (View Forum; Philips Medical Systems) to obtain both the maximum-intensity projection (MIP) and volume rendering (VR) images. Cervical MRA was performed using the high spatial resolution CEMRA sequence with a 16-channel craniocervical joint coil. Parameters were as follows: a 4.7/1.79 msec TR/TE; 27° flip angle; 320 × 320 mm FOV; 150 slices; 1.0 mm slice thickness, matrix 704 × 704 and an acquisition time of 1 min 27 s. A 1 ml bolus dose of gadolinium (0.5 mol/l, Magnevist; Bayer Health Care Pharmaceuticals) was administered intravenously at a flow rate of 2.5 ml/s by a power injector, followed by 21 ml of saline flush to measure the time taken for the gadolinium to reach the aortic arch. Subsequently, 19 ml of gadolinium was injected at the same rate. The average scanning delay time was 13 s (11–19 s).

The intracranial portion of the internal carotid artery (I-ICA), the anterior, middle, and posterior cerebral arteries (ACA, MCA and PCA), the intracranial vertebral artery (I-VA), and the basilar artery (BA) were evaluated by intracranial MRA. The common carotid artery (CCA), the extracranial portion of the internal carotid artery (E-ICA), the extracranial vertebral artery (E-VA), the external carotid artery (ECA), and the subclavian artery (SUB) were evaluated by cervical MRA. All MRA findings were reviewed by two investigators who were blind to patient clinical data. The severity of arterial stenosis was rated into five grades depending on the narrowing of the arteries: without any reduction of arterial diameter; <10% reduction of arterial diameter; 10–50% reduction; 51–99% reduction; and complete occlusion [14]. If stenosis severity for a given artery was different between the right and left sides, the side with more severe stenosis was used for grade assignment and analysis. When two or more stenoses were detected in different arteries, the most severe arterial stenosis grade was used for patient classification.

### Ultrasound measurement

Common carotid arteries were assessed using a high resolution B-mode ultrasound (Sequoia 512, Siemens, Germany) equipped with a 10 MHz probe, as previously described [15]. A single sonographer blind to patient clinical characteristics measured bilateral carotid arteries. Both common carotid arteries were scanned from proximal to distal in relation to the bifurcation. IMT was measured at the far wall of both common carotid arteries, approximately 1 cm proximal to the carotid bulb. The carotid IMT value was calculated as the mean of the maximal IMT of each carotid artery.

### Anthropometric and biochemical measurements

Each patient had a physical examination including measurements of height, weight, and blood pressure in an air-conditioned, quiet room. We calculated body mass index (BMI) as weight (kg) divided by squared height (m). The blood pressure was measured indirectly using a mercury sphygmomanometer. Sitting blood pressure was measured after 5-min rest with a blood pressure cuff appropriately sized to arm circumference and placed on the subject’s non-dominant arm. The average of three measurements at two minute intervals was used for the analysis. Smoking status was based on an interview. Subjects were classified as nonsmokers or current smokers.

On a separate day from 3-day CGM measurement, venous blood sample was drawn on 6 AM after a 10 hour overnight fasting to test the biochemical measurements. Hepatic biomarkers, including alanine aminotransferase (ALT), aspartate aminotransferase (AST); renal function biomarkers including blood urea nitrogen (BUN), plasma creatinine, and uric acid; triglycerides (TG), total cholesterol (TC), high density lipoprotein cholesterol (HDL-C), and low density lipoprotein cholesterol (LDL-C), were determined by standard enzymatic methods using a biochemical analyzer (Hitachi 7600–020, Tokyo, Japan). Fasting plasma glucose levels were assayed by the glucose oxidase method. Hemoglobin A_1c_ (HbA_1c_) was measured by high-performance liquid chromatography with a Variant II Hemoglobin A_1c_ analyzer (Bio-Rad Laboratories, Hercules, CA, USA).

### Statistical methods

Statistical analyses were performed using SPSS software version 17.0 (SPSS Inc., Chicago, IL, USA). Normally distributed data are presented as mean ± SD, whereas skewed variables are presented as median (interquartile range: 25th to 75th percentile). Clinical characteristics that followed a normal distribution were compared among groups using one-way analysis of variance with post-hoc LSD test, while those with non-normal distribution were compared using the Kruskal–Wallis test followed by Mann–Whitney *U* test with Bonferroni correction. In addition, a chi-squared test was used to determine the differences between groups in categorical variables. Variables that did not follow a normal distribution were log-transformed. Logistic regression analysis was performed to identify independent factors for cervical and/or intracranial plaque formation. The results of the regression were expressed as odds ratios (OR) with 95% confidence intervals (CI). In patients with negative finding on MRA, Spearman correlation coefficients were employed for correlation analysis between carotid IMT and variables. Multiple regression models were used to explore the influence of different variables on carotid IMT and to adjust for covariates. We calculated the number of patients required for the study to reject the null hypothesis 90% of the time (i.e., with a 1-tailed type II error rate of 0.1) when r was 0.40 or higher with a 2-tailed type I error at the 0.05 level of significance. Because this calculation led to a sample size of at least 61, the number 63 of patients who had negative finding on MRA was sufficient. A *P* value of <0.05 (two-tailed) was considered to indicate statistical significance.

## Results

### Characteristics of study subjects

Subjects had a mean ± SD age of 63 ± 10 years (range: 40–86 years), mean HbA_1c_ levels of 8.3 ± 1.7 %, and a median (interquartile range) diabetes duration of 9.0 (5.0–13.3) years. Patients were classified according to the presence and severity of arterial stenosis: patients without stenosis (*n* = 63); <10% stenosis group: patients with less than 10% stenosis in the most severely stenotic artery (*n* = 105); 10–50% stenosis group: patients with 10–50% stenosis in the most severely stenotic artery (*n* = 40); 51–99% stenosis group: patients with 51–99% stenosis in the most severely stenotic artery (*n* = 8); no patient had complete occlusion on MRA. The baseline characteristics and laboratory data are shown in Table 1. 10-50% stenosis group and 51-99% stenosis group were combined as one group (10-99% stenosis group) listed in Table 1 because there were only 8 patients in the 51-99% stenosis group. Compared to patients without stenosis, patients in <10% stenosis group and 10-99% group were older and had significant higher TC levels. Also, patients in 10-99% stenosis group had significantly higher systolic blood pressure, MBG and LDL-C levels than the other two groups (all *P* < 0.05). However, SDBG and MAGE were not statistically different among the three groups (both *P >* 0.05).

**Table 1:** Clinical characteristics and medication use (%) at baseline of study participants

**Characteristics**	**Total**	**Without Stenosis**	**<10% Stenosis**	**10-99% Stenosis**^**a**^	***P*value**
	***n*** **= 216**	***n*** **= 63**	***n*** **= 105**	***n*** **= 48**	
Age, y	63 (10)	57 (9)	64 (9) ^*^	68 (10) ^*†^	<0.001
Gender, male/female	92/124	29/34	43/62	20/28	0.804
Body mass index, kg/m^2^	25.1 (3.8)	25.0 (4.3)	25.2 (3.5)	24.9 (3.7)	0.853
Diabetes duration, y	9.0 (5.0-13.3)	8.0 (5.0-13.0)	8.0 (4.0-14.0)	10.5 (8.5-12.8)	0.045
Systolic Blood Pressure, mmHg	135.0 (120.0-146.0)	125.0 (120.0-140.0)	135.0 (120.0-140.0)	140.0 (131.3-160.0) ^‡§^	<0.001
Diastolic Blood Pressure, mmHg	80.0 (75.0-85.3)	80.0 (70.0-80.0)	80.0 (70.0-85.0)	80.0 (80.0-93.8) ^‡^	0.030
HbA_1c_, %	8.3 (1.7)	8.2 (1.7)	8.3 (1.6)	8.7 (1.9)	0.186
MBG, mmol/L	8.9 (1.9)	8.4 (1.5)	8.8 (1.9)	9.6 (2.1) ^*†^	0.007
MAGE, mmol/l	5.6 (2.2)	5.5 (2.1)	5.7 (2.2)	5.6 (2.3)	0.800
SDBG, mmol/l	2.3 (0.8)	2.3 (0.8)	2.3 (0.8)	2.3 (0.9)	0.921
Fasting plasma glucose, mmol/l	7.8 (2.5)	7.6 (2.1)	7.7 (2.5)	8.4 (2.9)	0.266
Total cholesterol, mmol/l	4.7 (1.0)	4.4 (1.0)	4.8 (1.0) ^*^	5.1 (1.0) ^*^	0.002
Triglycerides, mmol/l	1.6 (1.0-2.2)	1.7 (1.0-2.4)	1.5 (1.0-2.2)	1.5 (1.1-2.2)	0.920
HDL-C, mmol/l	1.0 (0.9-1.2)	1.0 (0.9-1.2)	1.0 (0.9-1.3)	1.0 (0.9-1.2)	0.294
LDL-C, mmol/l	3.1 (0.8)	2.8 (0.8)	3.1 (0.8)	3.4 (0.8) *†	0.002
ALT, U/l	16.0 (12.0-26.0)	15.0 (12.0-27.0)	18.0 (13.0-26.5)	15.0 (11.0-26.0)	0.218
AST, U/l	18.5 (16.0-24.0)	17.0 (15.0-22.3)	20.0 (15.8-25.0)	19.0 (16.0-24.5)	0.199
BUN, mmol/l	5.5 (4.6-6.6)	5.1 (4.6-6.2)	5.7 (4.7-6.7)	5.7 (4.9-6.8)	0.141
Plasma creatinine, μmol/l	63.5 (54.0-81.0)	62.0 (53.0-76.0)	63.5 (53.0-81.0)	69.0 (54.5-84.5)	0.359
Uric acid, μmol/l	306.5 (257.0-374.3)	305.0 (265.0-393.0)	301.5 (246.0-357.8)	334.0 (267.0-384.5)	0.237
Smoking, n (%)	29 (13.4%)	14 (22.2%)	9 (8.6%)	6 (12.5%)	0.042
Medication use, n (%)					
Glucose control therapy ^b^					0.037
Oral hypoglycemic agents	68 (31.5%)	26 (41.3%)	33 (31.4%)	9 (18.8%)	
Insulin	96 (44.4%)	24 (38.1%)	42 (40.0%)	30 (62.5%)	
Oral hypoglycemic agents and insulin	48 (22.2%)	13 (20.6%)	27 (25.7%)	8 (16.7%)	
Lipid-lowering therapy	117 (54.2%)	30 (47.6%)	60 (57.1%)	27 (56.3%)	0.430
Statins	100 (46.3%)	22 (34.9%)	51 (48.6%)	27 (56.3%)	
Fibrates	17 (7.9%)	8 (12.7%)	9 (8.6%)	0 (0%)	
Antihypertensive therapy	139 (64.4%)	33 (52.4%)	69 (65.7%)	37 (77.1%)	0.025
ACE inhibitors	14 (6.5%)	3 (4.8%)	11 (10.5%)	0 (0%)	
Angiotensin II receptor blockers	109 (50.5%)	27 (42.9%)	49 (46.7%)	33 (68.8%)	
Calcium channel blockers	57 (26.4%)	9 (14.3%)	30 (28.6%)	18 (37.5%)	
Beta-blockers	15 (6.9%)	5 (7.9%)	6 (5.7%)	4 (8.3%)	
Aspirin use	108 (50%)	22 (34.9%)	58 (55.2%)	28 (58.3%)	0.016

Data are mean (SD), median (25th to 75th percentile) or n (%).

^a^ The group included 40 patients with stenosis between 10-50% and 8 patients with stenosis between 51-99%. No patient has occlusion on MRA.

^b^ Medication use during continuous glucose monitoring period; 4 patients received diet therapy.

^*^P <0.05 vs without stenosis group; ^†^*P* <0.05 vs <10% stenosis group; ^‡^*P* <0.01 vs without stenosis group; ^§^*P* <0.01 vs <10% stenosis group.

Abbreviations: *MBG*, mean blood glucose; *MAGE*, mean amplitude of glycemic excursions; *SDBG*, standard deviation of blood glucose; *HDL-C*, high density lipoprotein cholesterol; *LDL-C*, low density lipoprotein cholesterol; *ALT*, alanine aminotransferase; *AST*, aspartate aminotransferase; *BUN*, blood urea nitrogen; *ACE* inhibitor, angiotensin-converting enzyme inhibitor.

### MRA findings

MRA findings are summarized in Table 2. Of all subjects, 153 (70.8%) patients presented with at least one cervical and/or intracranial atherosclerotic lesion. The intracranial atherosclerotic lesions were localized mainly at PCA, I-ICA, ACA, MCA and the cervical atherosclerotic lesions were localized mainly at CCA.

**Table 2:** Magnetic resonance angiography findings

Stenosis rating^*^	**Intracranial**						**Cervical**				
	**I-ICA**	**ACA**	**MCA**	**PCA**	**I-VA**	**BA**	**CCA**	**SUB**	**E-ICA**	**ECA**	**E-VA**
Total	216	216	216	216	216	216	216	216	216	216	216
Without Stenosis	178	184	186	173	190	197	142	180	168	202	163
< 10% stenosis	28	29	15	30	17	15	68	34	38	12	48
10-50% stenosis	9	2	10	12	6	3	6	2	10	1	5
51-99% stenosis	1	1	5	1	3	1	0	0	0	1	0
Occlusion	0	0	0	0	0	0	0	0	0	0	0
Abnormal (%)	17.59	14.81	13.89	19.91	12.04	8.80	34.26	16.67	22.22	6.48	24.54

^*^Based on the rating of more affected side in case of bilateral vessel lesions.

Abbreviations: *I-ICA*, intracranial portion of the internal carotid artery; *ACA*, anterior cerebral artery; *MCA*, middle cerebral artery; *PCA*, posterior cerebral artery; *I-VA*, intracranial vertebral artery; *BA*, basilar artery; *CCA*, common carotid artery; *SUB*, subclavian artery; *E-ICA*, extracranial portion of the internal carotid artery; *ECA*, external carotid artery; *E-VA*, extracranial vertebral artery.

### Logistic regression analysis of the different risk factors for cervical and intracranial atherosclerosis

Of potential confounding risk factors, multiple logistic regression analysis revealed that age (OR 1.115; 95.0% CI 1.065–1.168), systolic blood pressure (OR 1.030; 95.0% CI 1.005–1.056), MBG (OR 1.245; 95.0% CI 1.000–1.549) and LDL-C (OR 1.633; 95.0% CI 0.971–2.746) were independent factors for the presence of cervical and/or intracranial lesions. The independent variables included age, sex, BMI, smoking status, diabetes duration, HbA_1c_, MBG, MAGE, SDBG, blood pressure, TG, HDL-C, LDL-C, ALT, AST, diabetes treatment, dyslipidemia treatment, antihypertensive treatment and aspirin use.

### Association between carotid IMT and glycemic variability

**Figure 1:**
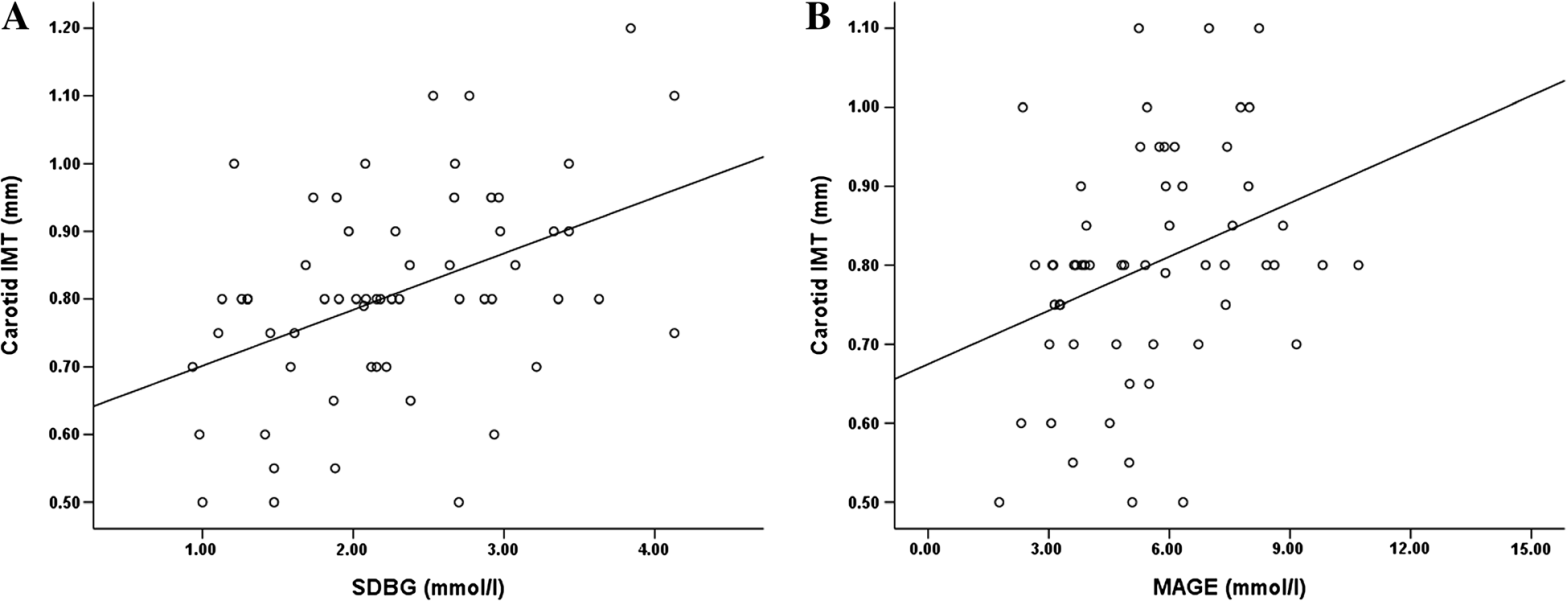
**Correlation between carotid IMT and glycemic variability in subjects without atherosclerotic lesion on MRA.** (**A**) The standard deviation of blood glucose (SDBG) was positively correlated to carotid IMT (*r* = 0.412, *P* = 0.001) (*n* = 63). (**B**) The mean amplitude of glycemic excursion (MAGE) was positively correlated to carotid IMT (*r* = 0.365, *P* = 0.005) (*n* = 63)

Patients were classified into 2 groups according to MRA results: subjects without plaque (*n* = 63) and subjects with plaque (*n* = 153). In the former group, when carotid IMT was tested for simple linear correlations against markers of glucose control and nonglycemic clinical and laboratory variables, statistically significant correlations were found with age (*r* = 0.497, *P* < 0.001), MAGE (*r* = 0.365, *P* = 0.005) and SDBG (*r* = 0.412, *P* = 0.001) (Figure 1). In contrast, in subjects with atherosclerotic plaque (*n* = 153), carotid IMT only correlated with age (*r* = 0.173, *P* = 0.038) among all the markers of glycemic and nonglycemic variables and no correlation was observed with MAGE (*r* = 0.089, *P* = 0.298) and SDBG (*r* = 0.122, *P* = 0.147).

Next, multiple linear regression analyses were performed to assess the independent effects of glycemic and nonglycemic variables on carotid IMT in patients without plaque (*n* = 63) (Table 3). The independent variables included age, diabetes duration, blood pressure, HbA_1c_, TG, HDL-C, LDL-C, MBG, SDBG and MAGE. Because a strong intercorrelation was observed between MAGE and SDBG (*r* = 0.813; ie, *R*^*2*^ =0.63) in the univariate analysis, 2 independent models were tested, 1 including the SDBG (model 1) and 1 the MAGE (model 2). Both SDBG (standardized β = 0.335, *P* = 0.005) and MAGE (standardized β = 0.319, *P* = 0.010) remained significant in stepwise regression analysis (multiple *R*^*2*^ = 0.314 for the model including SDBG and multiple *R*^*2*^ = 0.268 for the model including MAGE). The regression equations are as follows: Model 1: Carotid IMT (mm) = 0.270 mm + (0.007 mm * Age) + (0.065 mm *SDBG). Model 2: Carotid IMT (mm) = 0.272 mm + (0.007 mm * Age) + (0.023 mm * MAGE).

**Table 3:** Results of stepwise regression analysis with carotid IMT as the dependent variable in patients without lesions on MRA (n = 63)

**Explanatory variable**	**Standardized Regression Coefficient**	**t**	***P*Value**	**Adjusted*R***^***2***^**of the Model**
Model 1				0.314
Age	0.415	3.609	0.001	
SDBG	0.335	2.909	0.005	
Model 2				0.268
Age	0.413	3.470	0.001	
MAGE	0.319	2.682	0.010	

Abbreviations: *SDBG*, standard deviation of blood glucose; *MAGE*, mean amplitude of glycemic excursions.

## Discussion

The present study revealed a high prevalence of cervical and/or intracranial atherosclerotic lesions among type 2 diabetic patients as evaluated by MRA. More importantly, our study suggested that glycemic variability was associated with subclinical atherosclerosis before plaque formation because (1) SDBG and MAGE correlated with carotid IMT only in patients without atherosclerotic plaque; (2) conventional risk factors including elder age, hypertension, increased MBG and increased LDL-C were independent predictors of plaque formation; and (3) SDBG and MAGE were not significantly different among groups with different degrees of arterial stenosis.

### Studies on glycemic variability

Chronic hyperglycemia and glycemic variability are two major glucose characteristics of diabetes. The relationships between chronic hyperglycemia and chronic complications have been well-studied [16]. By contrast, the measure of glucose variability introduces the possibility that multiple fluctuations of blood glucose could be more dangerous than either chronic stable hyperglycemia or a simple episode of acute hyperglycemia. Compared to chronic hyperglycemia, glycemic variability is poorly understood, mainly due to limitations of glucose monitoring techniques. However, the emergence of CGM system has made it possible to record the complete picture of 24 h glucose excursions, and thus the glycemic variability can be accurately and conveniently calculated. Studies on glycemic variability have shown its association with postprandial ß-cell dysfunction [17] and glycemic variability is becoming an important parameter when evaluating the efficacy of different hypoglycemic treatments [18]. Moreover, glycemic variability is also strongly correlated to HbA_1c_ level in elderly male patients with type 2 diabetes [19], albeit glycemic variability does not provide additional prognostic value above and beyond already recognized risk factors for mortality during acute myocardial infarction [20]. On the contrary, the association between intraday glycemic variability and the presence and severity of coronary artery disease in patients with type 2 diabetes has been studied by Su et al. [21]. So far, the relationship between glycemic variability and diabetic macrovascluar complications remains unclear.

### Atherosclerosis detection: MRA and ultrasonography

Until now, few studies have addressed the association between glycemic variability and atherosclerosis. Chen et al. [22] studied 36 patients with type 2 diabetes and demonstrated that carotid IMT was correlated with MAGE. However, the authors used ultrasound to detect both preclinical atherosclerosis and the plaque formation. As a quantitative variable, carotid IMT is a feasible technique for evaluating subclinical atherosclerosis but may have several limitations when defining plaque, including inability to visualize intracranial vessels, difficulties in detecting near-total occlusion, and high reliance on operator skill [23,24]. For the Chinese population, assessing intracranial vessels is particularly important because East Asians were reported to be significantly more susceptible to intracranial atherosclerotic lesions than are Caucasians [25]. Thus, using ultrasound assessment alone, patients with only intracranial plaque cannot be accurately detected. Therefore, in terms of defining the presence of plaque, we evaluated both cervical and intracranial vessels using CEMRA and 3D-TOF-MRA. CEMRA is more specific and can provide additional diagnostic information than ultrasonography, while avoiding the potential complications of conventional angiography in assessing cervical vessels [7]. 3D-TOF-MRA allows more accurate evaluation of intracranial steno-occlusive disease among the noninvasive imaging techniques [8]. In addition, MRA technology depends less on the operator and is associated with lower inter-observer variability than is ultrasonography. To our knowledge, the present study is the first to use both MRA and ultrasonography to evaluate different stages of atherosclerosis in order to explore a potential relationship with glycemic variability.

### Glucose fluctuation and atherosclerosis

Using the CGM system we report that, in diabetic patients without atherosclerotic lesions, SDBG and MAGE were significantly related to carotid IMT, raising the intriguing possibility that glycemic variability plays a key role in the subclinical stage of atherosclerosis. Our results are partly in agreement with several *in vitro* and epidemiological studies suggesting that glucose fluctuation can harm the arterial wall through oxidative stress and/or endothelial dysfunction [4,5]. Meanwhile, as a major contributor to glycemic variability, postprandial blood glucose, especially the acute hyperglycemia after a meal or glucose load, was found to be strongly associated with endothelial function, carotid IMT and cardiovascular diseases [26,27]. Suzuki et al. [28] found the attenuation of brachial artery flow-mediated dilation (FMD) in the postprandial state was correlated with postprandial glucose fluctuation and the postprandial serum insulin level in individuals with normal glucose tolerance. Esposito, et al. [29] found that incremental glucose peak, the maximal incremental increase in blood glucose obtained at any point after the meal, was correlated with carotid IMT, suggesting postprandial glucose is involved in the process of subclinical atherosclerosis. In addition, the management of postprandial hyperglycemia in type 2 diabetes is related to regression of atherosclerosis [30]. For example, Mitiglinide, a short-acting insulinotropic agent to ameliorate postprandial hyperglycemia, was shown to reduce excess oxidative stress and inflammation [31].

### Hypothesis

We speculate that endothelial dysfunction may underlie the association between glycemic variability and subclinical atherosclerosis. Although atherosclerosis is commonly described as the presence of plaque obstructing the lumen of the conduit arteries, endothelial dysfunction is regarded as the initial step in the development of atherosclerosis and can be observed preceding structural atherosclerotic changes in the vascular wall or ultrasonic evidence of plaque [32,33]. Hashimoto et al. [34] studied the correlation between carotid IMT and FMD of the brachial artery, and found that FMD was related to carotid IMT only in subjects without overt atherosclerosis, which suggested that endothelial dysfunction correlates with carotid IMT before the development of macroscopic anatomic atherosclerosis. Therefore, because glycemic variability was reported to be associated with endothelial dysfunction [5], and endothelial dysfunction, the initial process of atherosclerosis, seems to be correlated with carotid IMT in subjects without overt plaque, we speculate that the association between larger glycemic variability and increased carotid IMT is most likely due to the mechanism of endothelial dysfunction. Unfortunately, we did not measure any marker to substantiate our assumption in this study. Further studies need to be carried out to elucidate the mechanism.

### Clinical significance

Our study revealed that glycemic variability is related to subclinical atherosclerosis before the plaque is formed. On the other hand, we identified conventional risk factors such as age, hypertension, dyslipidemia and increased mean blood glucose level as important independent predictive factors for atherosclerotic plaque formation instead of glycemic variability. Indeed, substantial evidence suggests that chronic hyperglycemia plays a specific role in atherosclerosis progression in patients with diabetes [35]. Needless to say, the combination of antihyperglycemic treatment with lipid-lowering, antihypertensive, and antiplatelet therapy is currently regarded as the primary strategy for reducing the burden of macrovascular complications in diabetes [36-38].

In light of these results, we highlight the importance of adopting a multifactorial approach to the prevention of macrovascular disease in type 2 diabetic patients. Identifying patients at risk for atherosclerotic lesions using MRA technique may be a priority. In patients with lesions, strict blood-pressure control, lipid-lowering therapy should be emphasized. For those who have negative finding by MRA, early initiation of glycemic variability management is likely to be beneficial in preventing the development and progression of atherosclerosis.

### Limitations

The present study has some limitations. First, even though all the included patients received stable therapeutic regimen for 3 months before the study, a 2-day CGM profile may not sufficiently reflect the actual glycemic condition over a longer period. Second, because this was a cross-sectional study, our conclusions are based essentially only on correlation analysis. A larger prospective investigation is required in order to further demonstrate the role that glycemic variability plays in the process of atherosclerosis. Third, we did not assess the relationship between endothelial dysfunction and glycemic variability in the study to substantiate our results. Further studies are needed in the future.

## Conclusions

In conclusion, glycemic variability is associated with subclinical atherosclerosis in type 2 diabetic patients. Further studies will be necessary to clarify the role of glucose variability as a potential contributory factor to the complex processes of atherosclerosis development.

## Abbreviations

CGM: Continuous glucose monitoring; IMT: Intima-media thickness; 3D-TOF-MRA: 3D time-of-flight magnetic resonance angiography; CEMRA: Contrast-enhanced magnetic resonance angiography; MBG: Mean blood glucose; SDBG: Standard deviation of blood glucose; MAGE: Mean amplitude of glycemic excursions; I-ICA: Intracranial portion of the internal carotid artery; ACA: Anterior cerebral artery; MCA: Middle cerebral artery; PCA: Posterior cerebral artery; I-VA: Intracranial vertebral artery; BA: Basilar artery; CCA: Common carotid artery; E-ICA: Extracranial portion of the internal carotid artery; E-VA: Extracranial vertebral artery; ECA: External carotid artery; SUB: Subclavian artery; BMI: Body mass index; ALT: Alanine aminotransferase; AST: Aspartate aminotransferase; BUN: Blood urea nitrogen; TG: Triglycerides; TC: Total cholesterol; HDL-C: High density lipoprotein cholesterol; LDL-C: Low density lipoprotein cholesterol; HbA_1c_: Hemoglobin A_1c_; FMD: Flow-mediated dilation.

## Competing interests

The authors declare that they have no competing interests.

## Authors’ contributions

YM, JZ, ML and WJ conceived and designed the study. XM, DL, WL and CH recruited samples, ML and ML reviewed all the MRA results, YM, JZ and ML wrote the first draft of the paper. YW and YB critically reviewed the paper and contributed to discussion, and all authors revised the manuscript for important intellectual content and have approved the final version.
